# Post-pregnancy osteoporosis-related multiple vertebral fractures associated with post-partum thyroiditis: A CARE-compliant case report

**DOI:** 10.1097/MD.0000000000027615

**Published:** 2021-10-29

**Authors:** Hyeong-Wook Han, Na-Mo Jeon, Jae-Min Lee, Jae-Hyung Kim

**Affiliations:** a Department of Rehabilitation Medicine, Catholic Kwandong University International St. Mary's Hospital and Catholic Kwandong University, College of Medicine, Incheon, Korea; b Department of Rehabilitation Medicine, Catholic Kwandong University International St. Mary's Hospital and Catholic Kwandong University, College of Medicine, Incheon, Korea.

**Keywords:** hyperthyroidism, osteoporosis, pregnancy, thyroid

## Abstract

**Introduction:**

Osteoporosis is a condition commonly observed in elderly and postmenopausal women. Pregnancy and lactation-induced osteoporosis are rare, and the development of severe vertebral fractures is uncommon. Postpartum thyroiditis (PPT) is a minor cause of osteoporosis. To the best of our knowledge, the development of osteoporosis associated with pregnancy has not yet been reported.

**Patient concerns:**

Here, we report a rare case of post-pregnancy osteoporosis-related multiple vertebral fractures associated with PPT. A 25-year-old woman developed lower back pain after her first delivery. She was then admitted to our medical center because of aggravated back pain.

**Diagnosis:**

On radiographic examination, she had multiple compressions of the lumbar spine. Bone mineral density was associated with osteoporosis. Laboratory tests, thyroid scans, and thyroid ultrasonography were performed. The patient was diagnosed with PPT.

**Interventions:**

The patient stopped lactating immediately. She was administered bisphosphate at 3 mg/3 months intravenously, elementary calcium at 1000 mg/day, and calcitriol 0.5 μg/day.

**Outcomes:**

A month later, her pain was relieved by proper management and she could independently walk indoors.

**Conclusion:**

PPT might play a role in aggravating post-pregnancy osteoporosis. It should be considered as a differential diagnosis in patients presenting with postpartum osteoporosis-related multiple spine fractures.

## 1 Introduction

Post-pregnancy osteoporosis is a rare clinical problem.^[1]^ Its prevalence is unknown and, furthermore, the etiology remains controversial.^[1,2]^ There are 3 types of post-pregnancy osteoporosis: transient osteoporosis of the hip in pregnancy, post-pregnancy spinal osteoporosis, and osteoporosis associated with lactation. There are various causes of post-pregnancy osteoporosis, with pregnancy and lactation known to be strong risk factors for osteoporosis.^[3]^ Some cases of post-pregnancy osteoporosis have been reported.^[4–7]^

The prevalence of postpartum thyroiditis (PPT) varies from 1.1% to <16.7%, with a mean prevalence of 7.5%.^[8]^ No study has reported the development of osteoporosis and/or increased fracture risk associated with PPT which is also a risk factor for osteoporosis.^[9]^ To the best of our knowledge, post-pregnancy osteoporosis-related multiple spinal fractures associated with PPT have not been reported. Here, we report a rare case of post-pregnancy osteoporosis associated with PPT.

## 2 Case report

A 25-year-old woman developed lower back pain after her first delivery. As time progressed, her lower back pain worsened. She was previously diagnosed with lower back sprain at another medical center and treated with physical modalities and analgesic myorelaxants. However, these treatments failed to provide pain relief. The patient was admitted to our medical center because of severe back pain after collapsing to the ground a month after delivery. She was unable to ambulate or transfer because of severe back pain, and also experienced difficulties with her daily activities. Due to her severe pain, the patient was unable to hold or breastfeed her baby so she used a breast pump for lactation. The patient reported that she had been completely healthy before pregnancy. She had no diseases that could cause osteoporosis and did not use any medications. She was a nonsmoker and had no family history of osteoporosis or thyroid disease. She weighed in at 47 kg with a height of 163 cm. Informed written consent was obtained from the patient for publication of this case report.

On physical examination, tenderness was observed in the thoracolumbar vertebrae and spasm of the vertebral muscles. Although her spinal range of motion was limited, there were no abnormal neurological symptoms or signs on physical examination. The pain was localized to the back. There was no numbness or weakness.

**Figure 1:**
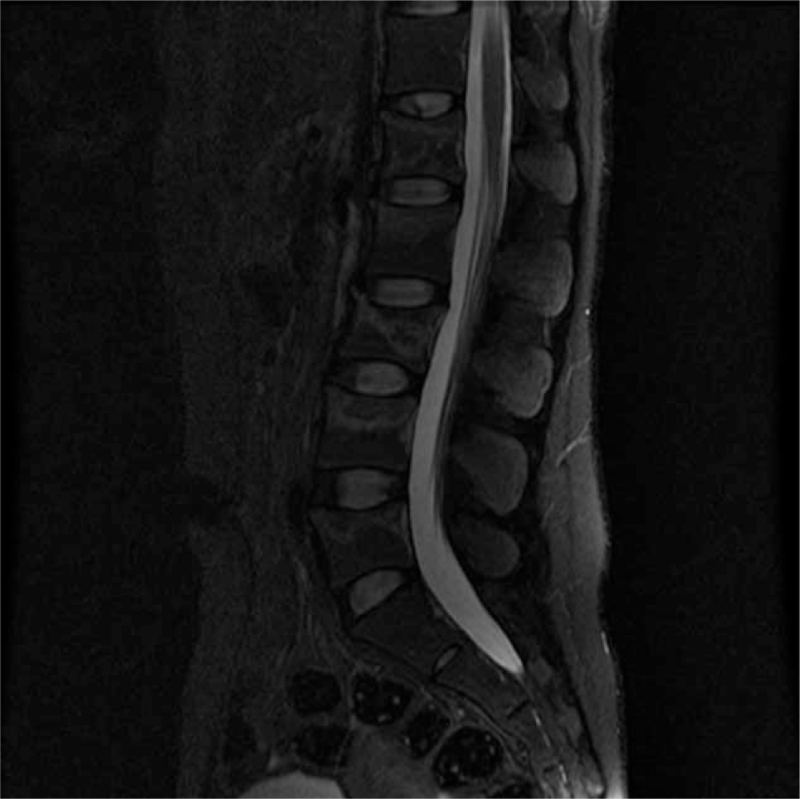
T2-weighted lumbar spinal magnetic resonance image showing trabecular compression fracture, multiple vertebrae (L1–5), intact posterior elements, no bulging disk, and no abnormal signal in cord.

**Figure 2:**
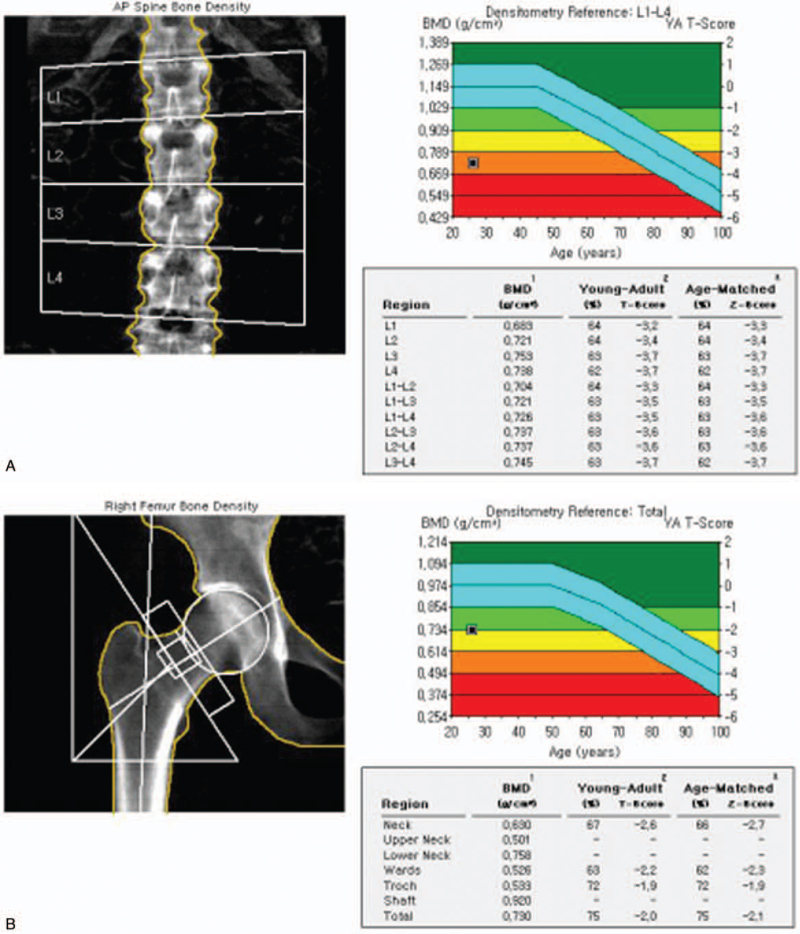
Bone mineral density measured for lumbar spine (A) and femur (B) by dual-energy x-ray absorptiometry. Findings were compatible with osteoporosis.

A bone scan showed multiple compressions of the lumbar spine, consistent with the lumbar magnetic resonance image (Fig. 1). Bone mineral density showed that the *t* score of the L1–4 spine was −3.5 and the *z* score −2.5 (Fig. 2A). Both the *t* score and the *z* score of the femur neck were −2.6 (Fig. 2B). In the thyroid scan, decreased trapping was observed in both thyroids (Fig. 4). On thyroid ultrasonography, no abnormal nodules were detected.

**Figure 3:**
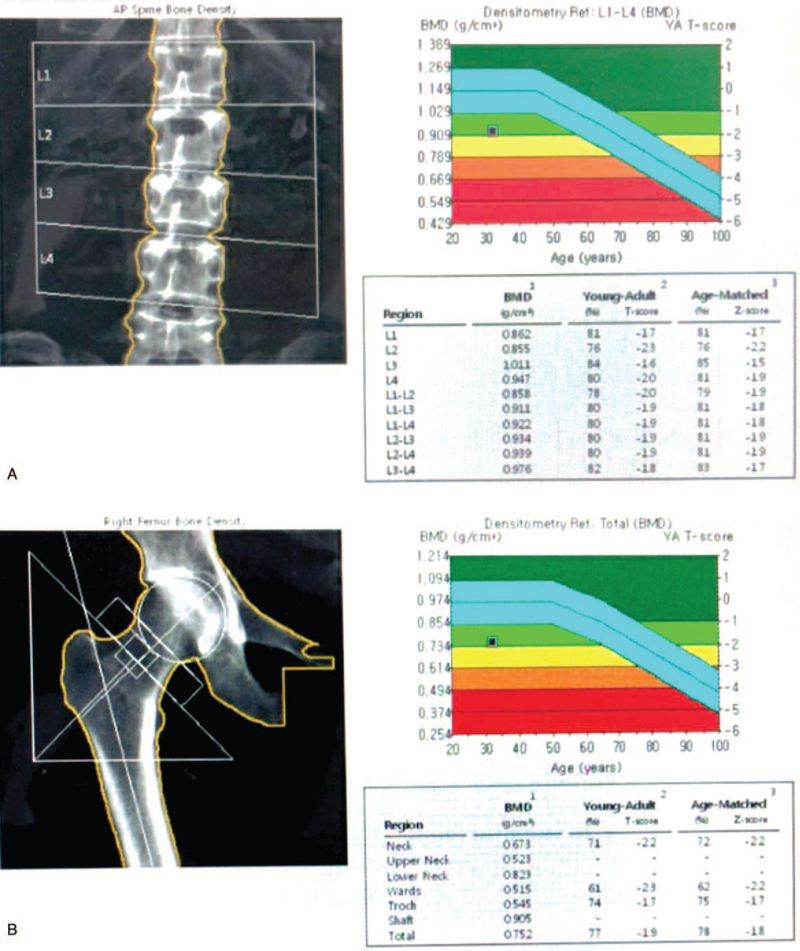
Bone mineral density measured for lumbar spine (A) and femur (B) by dual-energy x-ray absorptiometry after long-term follow-up.

**Figure 4:**
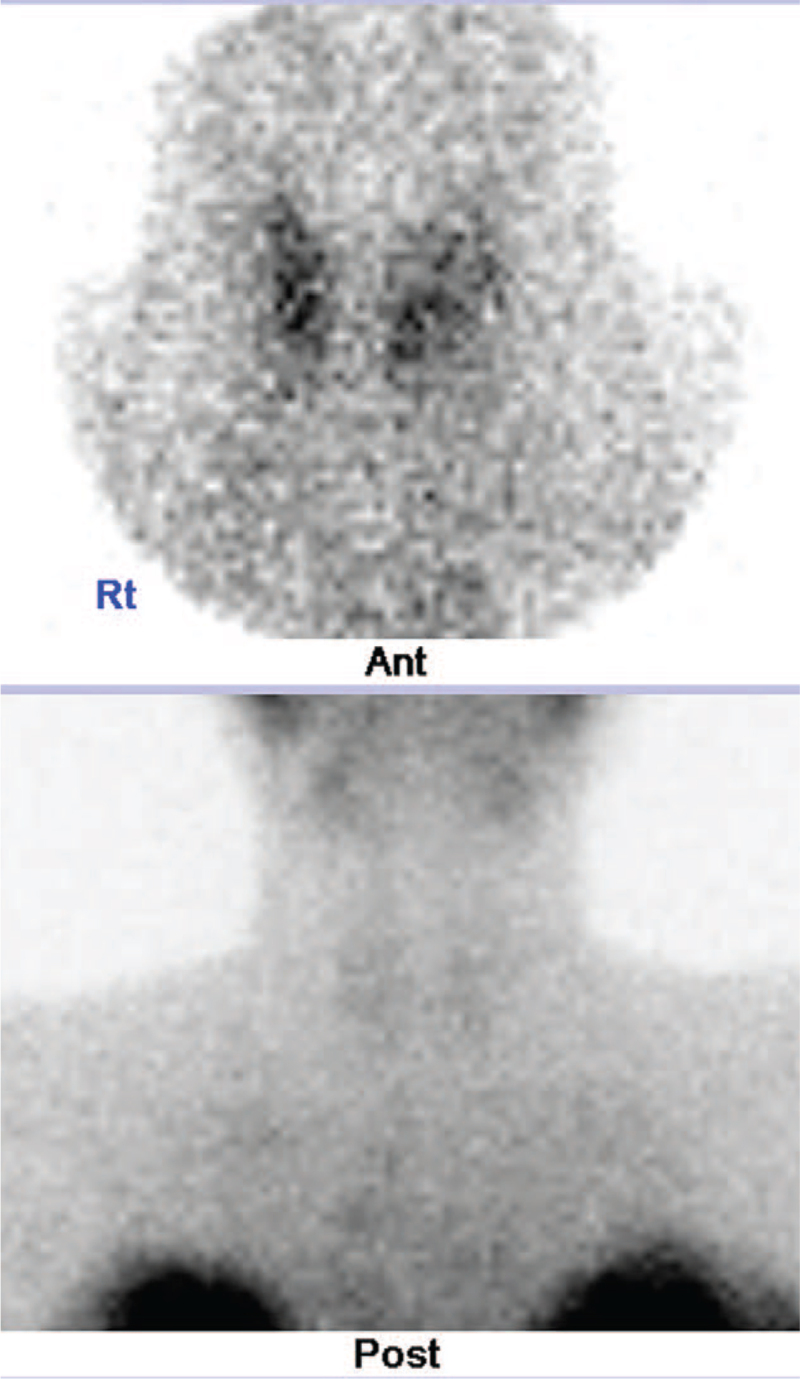
Thyroid scan showing no significant radioactive iodine uptake.

The laboratory findings showed a total calcium level of 10.4 mg/dL (normal range: 8.1–10.4), plasma 25-OH Vit D level of 9.2 ng/mL (normal range: 4.8–52.8), intact parathyroid hormone (PTH) level of 31.1 pg/mL (normal range: 14.0–72.0), PTH-related peptide level of 1.1 pmol/L (normal range: 0–1.1), adrenocorticotropic hormone level of 19.7 pg/mL (normal range: 5.0–60.0), cortisol level of 7.31 μg/dL (normal range: 3.09–16.66), and erythrocyte sedimentation rate of 11 mm/hr (normal range: 0–25). However, there were increases in the alkaline phosphatase level at 4.7 mg/dL (normal range: 2.7–4.7), T3 level at 253 ng/dL (normal range: 65–150), free T4 level at 2.94 ng/dL (normal range: 0.78–1.54), thyroid microsomal Ab level at 1300 U/mL (normal range: 0.0–60.0), prolactin level at 90.20 ng/mL (normal range: 4.79–23.30 ng/mL), and estrogen level of 265 pg/mL (normal range: 61–437 pg/mL). In addition, the TSH level at 0.04 μIU/mL (normal range: 0.35–5.50) and progesterone level at 0.4 μg/mL were decreased.

The patient was advised to immediately discontinue lactation. In addition, a thoracolumbosacral orthosis was prescribed. A bisphosphate intravenous injection of 3 mg/3 months, elementary calcium at 1000 mg/day, and calcitriol at 0.5 μg/day were commenced. For pain management, analgesics and physical modalities were recommended. The patient underwent a lumbar facet block. An exercise program including back muscle strengthening, range of motion, and relaxation exercises, as well as weight-bearing exercises, was started. A month after rehabilitative management, her pain improved (visual analog scale score improved from 6 to 2). She was able to walk independently indoors. At the 3-month follow-up, she had only L4–5 focal area back pain, felt better, and was comfortable performing daily activities. There was no new vertebral fracture or progressive collapse of pre-existing vertebrae. She received antiosteoporosis treatment (ibandronate intravenous injection 3 mg/3 months, elementary calcium 1000 mg/day, calcitriol 0.5 μg/day) during the rehabilitation process. About 5 years later, the following measurements were found to be within normal range: total calcium level of 9.6 mg/dL (normal range: 8.1–10.4), intact PTH level of 30.4 pg/mL (normal range: 14.0–72.0), plasma 25-OH Vit D level of 12.1 ng/mL (normal range: 4.8–52.8), alkaline phosphatase level at 3.2 mg/dL (normal range: 2.7–4.7), T3 level at 119 ng/dL (normal range: 65–150), free T4 level at 1.47 ng/dL (normal range: 0.78–1.54), and TSH level at 1.05 μIU/mL (normal range: 0.35–5.50). Finally, the BMD score improved. It showed that the *t* score of the L1–4 spine was −1.9 and the Z-score was −1.8 (Fig. 3A). The *t* score and the *z* score of the femur neck were both −2.2 (Fig. 3B).

## 3 Discussion

Several studies have reported post-pregnancy osteoporosis-related multiple spinal fractures.^[10]^ The mechanisms of pregnancy and lactation-associated osteoporosis are suggested as follows. The developing fetal skeleton accretes approximately 30 g of calcium by term. Approximately 80% of the cases occur during the third trimester. Approximately 280 to 400 mg of calcium is lost daily through breast milk, whereas a female who is nursing twins loses approximately 1000 mg of calcium per day. Pregnancy and lactation can cause fragility fractures in women due to osteoporosis.^[11]^

In our case, a 25-year-old previously healthy woman suffered multiple spinal fractures post-pregnancy. Laboratory findings showed an increase in the alkaline phosphatase levels. The total calcium and PTH-related peptide levels were within the upper normal limit range. These findings were compatible with those of lactate-induced osteoporosis. According to Karlsson et al, pregnancy and lactation are not risk factors for osteoporosis or fractures because of adaptations of mineral metabolism in most normal and healthy pregnant and lactated women.^[12]^ However, in rare cases, if this adaptation of mineral metabolism is not induced, osteoporosis can develop. In the present case, although the patient was breastfeeding after pregnancy, osteoporosis-related multiple spine fractures were more severe than expected. Thus, PPT might be a factor that could aggravate post-pregnancy osteoporosis. In other words, a combination of pregnancy lactation and an additional risk factor such as PPT for increasing fracture fragility may result in more severe osteoporosis and vertebral fractures.

We immediately stopped lactation and performed anti-osteoporotic therapy in patients diagnosed with PPT and post-pregnancy osteoporosis as a previous study.^[4]^ In addition, we implemented rehabilitation treatment programs, such as applying spinal orthosis and core muscle strengthening exercises. After the rehabilitation program, the pain caused by the fractured vertebrae was improved, along with the functional state, such as daily activities and gait. As a result of the long-term follow-up study, the thyroid function test recovered normally, and the BMD test results showed improvement of osteoporosis.

The patient presented with a painless thyroid gland, with no evidence of inflammation. Based on clinical findings, laboratory findings, and imaging studies, we ruled out subacute thyroiditis. She was diagnosed with PPT (ongoing hyperthyroidism). Postpartum thyroiditis is not a rare condition during pregnancy.^[8,13]^ Its clinical course may involve hyperthyroidism, hypothyroidism, or the 2 sequentially.^[13]^ Both hyperthyroidism and hypothyroidism are known risk factors for osteoporosis.^[14]^ Recent studies have reported that endogenous subclinical hyperthyroidism can decrease bone mineral density due to high bone turnover. Finally, hyperthyroidism and hypothyroidism can cause osteoporotic fractures.^[4,15]^ The PPT might have increased the risk of fragility fracture. However, the association of PPT with fractures has not yet been reported. Generally, PPT does not suffice to cause osteoporotic fracture because this disease is transient. However, in our case, pregnancy and lactation-induced osteoporosis overlapped with PPT, which might have increased the risk and severity of osteoporosis. Many factors are responsible for osteoporotic fractures and the PPT may have served as a potential factor that aggravated osteoporosis. Therefore, PPT should be considered when treating patients with postpartum osteoporosis-related multiple spine fractures.

## 4 Conclusions

This is a novel case report of multiple spine fractures in a young woman with PPT and pregnancy osteoporosis. The PPT might have played a role in aggravating post-pregnancy osteoporosis. A careful diagnostic approach should therefore be considered when young patients present with postpartum osteoporosis-related multiple spine fractures.

## Acknowledgments

The authors thank Editage (www.editage.co.kr) for English language editing.

## Author contributions

**Conceptualization:** Hyeong-Wook Han, Na-Mo Jeon, Jae-Min Lee, and Jae-Hyung Kim.

**Resources:** Jae-Min Lee, Jae-Hyung Kim.

**Supervision:** Jae-Hyung Kim.

**Writing – original draft:** Hyeong-Wook Han.

**Writing – review & editing:** Na-Mo Jeon, Jae-Hyung Kim.
